# Modern methods and materials used to treat root perforation: effectiveness comparison

**DOI:** 10.1007/s10856-023-06770-y

**Published:** 2024-01-11

**Authors:** XiaoLan Ma, Hua Xu, Xuefang Chen, Qian Zou, Junrong Wang, Yunmeng Da, Huisu Yin

**Affiliations:** 1 Department of Oral Medicine, Hebei Eye Hospital, 399 East Quanbei Street, Xingtai, 054001 China; 2 Department of Oral and Maxillofacial Surgery, Hebei Eye Hospital, 399 East Quanbei Street, Xingtai, 054001 China; 3 Department of Prosthodontics, Hebei Eye Hospital, 399 East Quanbei Street, Xingtai, 054001 China; 4 Department of Science and Education, Hebei Eye Hospital, 399 East Quanbei Street, Xingtai, 054001 China

## Abstract

This study aims to experimentally compare the efficacy of different endodontic materials (iRoot BP Plus, Biodentine, MTA, Rootdent, and Trioxide) in the treatment of pulpitis and perforations on extracted tooth specimens. Additionally, the study aims to investigate the influence of iRoot BP Plus endodontic material on the regenerative processes following pulp amputation in laboratory animals. The secondary goal is to evaluate the effect of iRoot BP Plus on the restoration process in laboratory animals after pulp removal. The study presents a micropermeability analysis of the selected biomaterials performed on a sample of 50 single-rooted apical teeth in 2022. All teeth underwent endodontic treatment. Changes in molar morphology were investigated with eight laboratory animals (rabbits, 3 months old, all males) after simulated pulp removal and subsequent treatment with the iRoot BP Plus biomaterials. iRoot BP Plus appeared to be more effective in retrograde apical root filling than other biomaterials, as evidenced by its higher sealing effect. An experiment involving animal participants revealed the presence of protective adaptive mechanisms, which manifested in the form of an inflammatory process within 6 weeks after the dental pulp was removed. The connective tissue replaced the necrosis, and new capillaries began to form intensively. These dental outcomes suggest that iRoot BP Plus enables hermetical sealing in tooth restoration with good adhesion. Thus, it may have the ability to promote more active tissue regeneration after pulp removal.

**Graphical Abstract:**

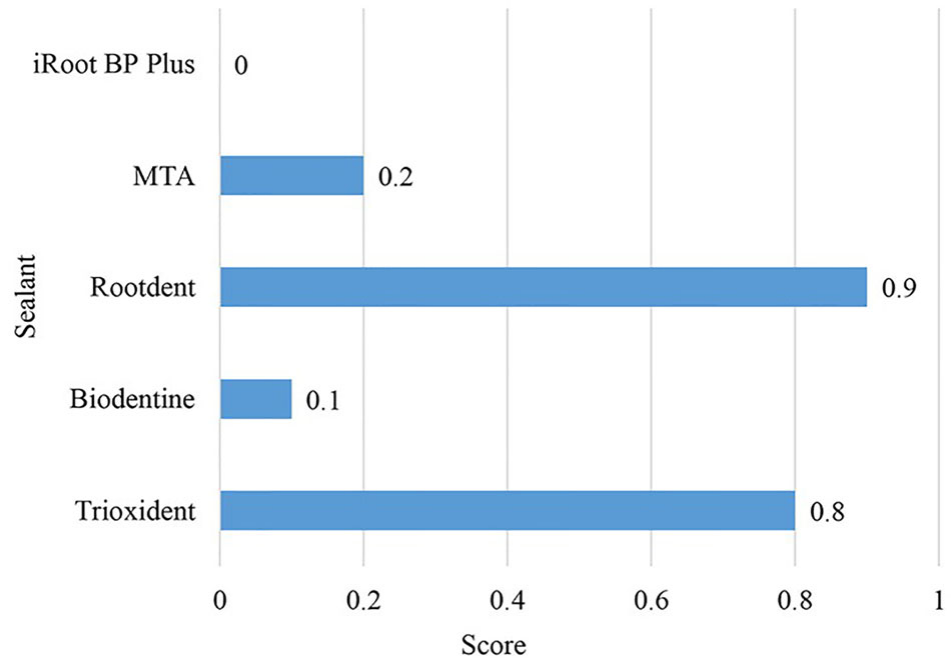

## Introduction

Dental caries is a rather common infectious disease, with the onset of allergic processes, weakening of the immune system, and prolonged duration of illness that affects other organ systems. Pulpitis is one of the most prevalent dental diseases, especially among children. In pediatric dentistry, it represents 30–40% of dental cases [1]. Therefore, dental pulp treatment should be done with caution, especially if tooth structures are still developing and may show inadequate responses to stimuli. The dental pulp is an important part of the tooth, as it provides root formation, growth, and resorption. Some of the methods used in dental pulp treatment include indirect procedures, direct pulp capping, and pulpotomy [2].

Pulp therapy involves a wide variety of endodontic biomaterials. The traditional one is calcium hydroxide. Yet, it has become less popular as some authors report a triggering effect of calcium hydroxide on the tooth resorption process, especially in patients with temporary teeth. It contains compounds that activate the mesenchymal dental pulp cells, which participate in root resorption. In addition, calcium hydroxide can be mechanically unstable and leak at the microscopic level [3], creating a window for microorganisms to penetrate the hard tissues and cause secondary inflammation directly in the pulp [4]. Some clinical studies reported a 95–75% success rate for calcium hydroxide pulpotomy 1 and 3 years after the intervention, respectively [5]. At the same time, other authors indicate lower success rates – 50 to 87% of success in 2 years [6]. A histological study shows that calcium hydroxide is responsible for the emergence of necrotic foci and inflammation areas [7]. In addition, tunnel defects were reported that depended on the pH value of the filling material.

A good alternative to calcium hydroxide is materials based on bioceramics. These materials are used in the treatment of pulpitis and dental canals. The bioceramic endodontic biomaterials are made up of aluminum, zirconium, and glass with biologically active properties [8]. Bioceramics are non-toxic, do not shrink, and are chemically stable. When solidifies, bioactive ceramic material acquires a hydroxyapatite coating. The most commonly used bioceramic endodontic biomaterials are mineral trioxide aggregate, Bioaggregate, Biodentine, and iRoot [9].

### Literature review

MTA, or mineral trioxide aggregate, is the first bioceramic endodontic biomaterials used in dental practice. In addition to water, the material hydrates to form calcium silicate hydrate gel with CaO crystals responsible for alkaline reactions. During hardening, MTA releases Ca ions, which subsequently enter dentinal tubules, thus increasing the concentration of Ca ions in the dentin. The shortest sitting time of MTA is 3 h [10]. The drawbacks of MTA include a long sitting time, high cost, and potential for discolouration.

Given the drawbacks of MTA, there is a need to develop other filling materials based on bioceramics. The novel bioceramic materials feature high biological activity, low toxicity to tissues and cells, and non-inflammation. These properties can stimulate dental tissue regeneration [11]. The use of bioceramic endodontic biomaterials promotes osteoinduction. In addition, these materials have proven to be highly effective in pulp manipulations and perforations [12]. One of the commonly used endodontic biomaterials is Biodentine. Its physicochemical properties are better than those of MTA. In particular, it can be applied as dentin replacement. Hence, this biomaterial is often used in the treatment of pulp diseases. Biodentine was reported to exhibit marginal adaptation and prevent bacterial leakage [13, 14] and tooth root displacement (if the applied paste layer is 4 mm thin). Because of these properties, Biodentine has been advocated for use in retrograde filling. At the same time, Biodentine can be soluble and have a low sealing ability in an acidic environment [15]. Other disadvantages include low radiopacity and low washout resistance. Since it does not contain fluoride anion, the material also has reduced antibacterial properties. According to some reports, Biodentine can lead to the formation of apatite if combined with ionomer glass [7].

Another novel biomaterial which has recently appeared on the market is iRoot BP/BP Plus (Innovative Bioceramics, Canada) [16]. These biomaterials are calcium silicate materials intended for root canal fillings and root perforations. Judging from the manufacturer’s instructions, iRoot BP/BP Plus are not inferior to MTA. However, some studies report that iRoot BP/BP Plus have a lower sealing ability compared to MTA [11]. At the same time, these endodontic biomaterials are easy to use and have two times shorter sitting time than MTA (about 2 h). Besides iRoot BP Plus, the manufacturer offers an iRoot FS sealer reinforced with nanoparticles [15]. This biomaterial has the same application scope, but it hardens, hydrates, and shrinks faster than MTA [17]. Another filling material of this class is iRoot SP. It comes in a ready-to-use form and can be used to seal root canals. iRoot SP can penetrate the dentinal tubules, thereby creating a strong bond with the dentin. This ability comes from its considerable penetration area, which is higher compared to other biomaterials, even though its adhesion potential is lower compared to the “classic” MTA [9]. iRoot SP not only has an excellent apical sealing ability, but it becomes even better when the root canals are moist. At the same time, apical seals will last longer if the root canal is dry upon the insertion of iRoot SP. Based on the foregoing, bioceramic materials can serve as dentin replacements and exhibit antibacterial properties, sufficient expansion coefficient, good adhesion properties, and an ability to harden in moist root canals [10]. Hence, calcium silicate-based filling materials can be deemed an excellent choice for creating a favorable environment for tooth regeneration [18].

Moreover, composites based on silver nanoparticles, zinc(II), nano-diamonds, and others can find applications in dental therapy, endodontics, orthodontics, periodontology, implantology, maxillofacial surgery, and denture prosthetics. However, it is noted that materials containing hydroxyapatite and bioactive glass nanoparticles may have limited effectiveness in certain dental areas [19]. Research [20] presents the development of a modifier based on cyclophosphazenes with 4-allyl-2-methoxyphenoxy and β-carboxyethylphenoxy, intended for blending with acrylic dental restorative compositions. It was found that these compositions exhibit improved adhesion to dental tissues, polymerization depth, as well as reduced water absorption and solubility. Elasticity, compressive strength, and microhardness values also increased with the increased content of the modifier in the composition [20]. Thus, the spectrum of applicable composites is quite broad, necessitating comparative studies of these compositions.

### Problem statement

In light of the considerable prevalence of endodontic biomaterials available in the marketplace, an assumption may arise that evaluating their necessity is unnecessary. However, the comparative effectiveness of different endodontic biomaterials, with their advantages and limitations, is a running issue in modern dentistry. Some of the research gaps include biocompatibility, adhesion, and tightness of endodontic biomaterials [21]. Different sealing agents may exhibit different properties under different humidity conditions. In addition, some endodontic biomaterials arrive in a ready-to-use form, while others need to be prepared beforehand. Other requirements that comparative studies have overlooked are the toxicity and antibacterial properties of sealing materials [22, 23]. The use of various endodontic biomaterials during pulp amputation also remains debatable [3, 7, 12]. This study seeks to narrow this gap by comparing the novel root canal sealer iRoot BP Plus with other endodontic biomaterials.

This study aims to experimentally compare the efficacy of various endodontic materials (iRoot BP Plus, Biodentine, MTA, Rootdent, and Trioxide) in the treatment of pulpitis and perforations using extracted tooth specimens. Additionally, the study seeks to investigate the impact of iRoot BP Plus endodontic material on regenerative processes following pulp amputation in laboratory animals. The null hypothesis of the research is as follows: There are no statistically significant differences in the effectiveness of various endodontic materials (iRoot BP Plus, Biodentine, MTA, Rootdent, and Trioxide) in treating pulpitis and perforations on extracted tooth specimens, and there is no influence of iRoot BP Plus endodontic material on regenerative processes following pulp amputation in laboratory animals.

The formulated null hypothesis assumes that all the investigated endodontic materials have equal efficacy in the treatment of pulpitis and perforations, and there is no influence of the endodontic material iRoot BP Plus on regenerative processes following pulp amputation in laboratory animals. Therefore, if the *p*-value obtained during the statistical analysis of the data is less than the significance level (e.g., *p* ≤ 0.05), the null hypothesis will be rejected in favor of the alternative hypothesis, which posits the presence of statistically significant differences in the effectiveness of various endodontic materials and/or the impact of iRoot BP Plus endodontic material on regenerative processes following pulp amputation.

The investigation aims to:

1. compare the given endodontic biomaterials in terms of marginal permeability in the apical part of the tooth;
2. determine whether iRoot BP Plus can be used in dental pulp removal.

## Materials and methods

The study took place in 2022 in a dental clinic in Beijing (PRC). The research process consists of two phases that match the objectives of the study. Phase 1 was to evaluate the marginal permeability in extracted teeth sealed with different endodontic materials. Phase 2 was an experimental study performed on rabbits; the goal was to identify the effects of different endodontic biomaterials.

**Fig. 1:**
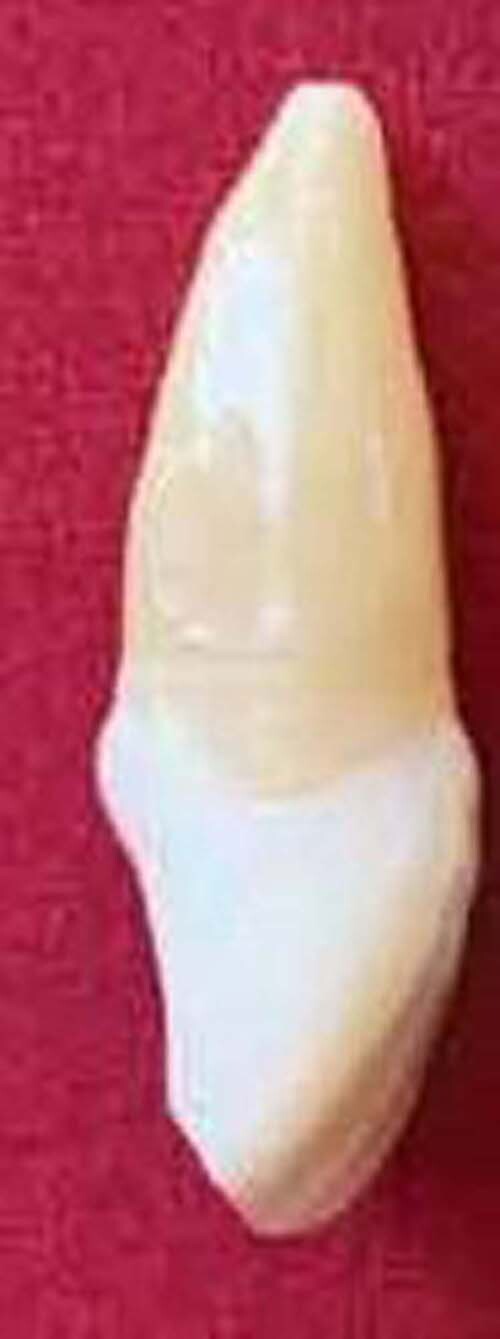
Sample of a single-rooted tooth extracted for permeability study

Phase 1 (in vitro) sample comprised 50 extracted single-rooted teeth (Fig. 1) sealed by five different endodontic biomaterials, namely Trioxident (VladMiVa, Russia), Biodentine (Septodont, France), Rootdent (TehnoDent, Russia), ProRoot MTA (Dentsply Endodontics, USA), and iRoot BP Plus (Innovative Bioceramics, Canada).

All teeth underwent endodontic therapy. The working length of each canal was evaluated using a #15 K-file (distance to the apical foramen was ≤0.5 mm). The apical part of the root canal was prepared with a #40 K file. The remaining space of the canal was treated using the conventional step-back technique. A 1% solution of chlorhexidine was used as an antiseptic. After cleaning, root canals were dried with paper points and then obturated with gutta-percha and root canal sealer using lateral condensation. Subsequently, the access to the root canal was blocked with glass-ionomer cement. Treated teeth were stored at room temperature and 100% humidity. The apical resection procedure was conducted afterwards, through which the top of the tooth was removed. The resection was made at 3 mm from the apex with a fissure bur at an angle of 90 degrees to the long axis of the tooth. The outer surface of the root was covered with two layers of varnish, except the apical 3 mm. The teeth were randomly divided into five groups of ten teeth each. The endodontic filling material was prepared according to the manufacturer’s instructions. The biomaterials were taken to the prepared root end cavity using a plugger. Each tooth was then immersed in a fuchsin solution for 1 day and washed under a stream of running water afterwards.

Two staining methods, van Gieson and Mallory, were employed for staining. Van Gieson staining method (penetration of the dye into treated teeth):

- Preparation of the dye: A 1% solution of fuchsin was prepared in distilled water.
- Preparation of the samples: Teeth intended for staining were prepared following the experimental protocol. The samples were clean and ready for dye application.
- Staining: The fuchsin solution was applied to the samples, ensuring even coverage. The dye was allowed to permeate the samples.
- Removal of excess dye: Following staining, the samples were thoroughly rinsed with distilled water to eliminate any excess dye and halt the staining process.
- Processing and Analysis: Upon completion of the staining procedure, the samples underwent essential processing, and the penetration of the dye into the materials was analyzed using microscopy with appropriate magnification.

Staining with Mallory’s method (tissue evaluation):

- Preparation of the dye: A solution of fuchsin was prepared at the optimal concentration for Mallory’s method (1%).
- Sample preparation: Samples for staining, such as tissue sections or specific tooth structures, were prepared for investigation.
- Staining: The samples were immersed in the fuchsin solution, allowing the dye to penetrate the tissues.
- Removal of excess dye: Following staining, the samples were rinsed to eliminate any excess dye and halt the staining process.
- Processing and analysis: After the completion of staining, the samples underwent necessary processing, and microscopic analysis of dye penetration into the tissues was conducted.

For the application of the dye and to achieve consistent results, “Microbrush” micro applicators (Microbrush International, USA) were utilized.

The degree of dye penetration was determined with a stereomicroscope at 10x magnification. All dye penetration measurements were conducted by the following test.

Dye Penetration Test in Dentistry: This test is employed to assess the infiltration of dye into the tooth structure or restorative materials.

For this a 3-point Dye Penetration Test was used where “0” means no dye penetration, “1” indicates dye penetration extending up to half of the interface between the biomaterials and tooth structure (marginal penetration) or into the filling material, and “2” represents dye penetration beyond half of the filling-tooth interface (complete penetration) [24]. The normal result using this scale is the absence of staining (0 points) or slight dye penetration not exceeding half of the sample (1 point). However, greater dye penetration (2 points) may indicate less effective hermetic sealing or the presence of issues in endodontic treatment. Ideally, when using an endodontic sealer, the best seal is expected to prevent dye penetration and potential reinfection of the root canals.

Phase 2 (in vivo) sample comprised eight male rabbits, all 3 months old. The animals underwent general anesthesia with 0.4 ml of ketamine (5%) per kilogram of body weight. The hard dental tissues were prepared by a handpiece with a micromotor. The opening and expansion of the pulp chamber were followed by the removal of the pulp crown. The root canal orifice was enlarged with the help of a drill. The wound treatment was performed using sodium hypochlorite and caproic acid (5%). The cavity was then dried, sealed with iRoot BP Plus, and filled with glass-ionomer cement. 6 weeks after the procedure, treated teeth were fixed in a formalin solution (10%) and then decalcified. The resulting histological samples were subjected to examination.

### Study design and ethical issues

This study pertains to the application of agents in the field of dentistry and endodontics. It is experimental research conducted on animals (rabbits) to investigate the effectiveness of various endodontic sealers, such as iRoot BP Plus, in the processes of dental tissue regeneration and restoration after pulp amputation. Such investigations enable the examination of the mechanisms of action of these agents and their potential clinical applications in dentistry. Animal experiments represent a crucial stage in comprehending the efficacy and safety of the agents before their potential implementation on patients in clinical settings.

Used in this study were extracted teeth obtained from a dental clinic. The utilization of biomaterials, such as endodontic sealers, was driven by the aim of this research to investigate and compare different materials for effective pulp capping and treatment of perforations. The materials were prepared for experimentation following standard protocols and adhering to the manufacturers’ recommendations. Before the experiments, five different types of endodontic sealers were prepared: Trioxident (Russia, manufactured by VladMiVa), Biodentine (France, manufactured by Septodont), Rutdent (Russia, manufactured by TehnoDent), ProRoot MTA (USA, manufactured by Dentsply Endodontics), and iRoot BP Plus (Canada, manufactured by Innovative Bioceramics). The primary components of the water-soluble dental material “Trioxide” are calcium, silicon, and aluminum oxides. The dental material “Bioceramic-based sealer” is an insoluble radiopaque material based on calcium silicate, free from aluminum, and set in the presence of moisture. Rutdent incorporates calcium, silicon, and aluminum oxides, with the addition of zirconium oxide for radiographic visibility. MTA material contains tricalcium silicates, aluminates, oxides, as well as silicate oxide with a small content of other mineral oxides, including bismuth oxide responsible for radiopacity. The composition of iRoot BP Plus includes calcium silicates, zirconium oxide, tantalum pentoxide, calcium sulfate (anhydrite), monocalcium phosphate, and fillers.

To ensure standardized application conditions for the materials, requirements about proportions, mixing, and consistency of each endodontic sealer were meticulously observed.

The application of materials in the experiments was conducted in two stages (in vivo and in vitro), strictly adhering to predetermined protocols. In the first part of the study, endodontic therapy and subsequent canal obturation were performed on 50 extracted single-rooted teeth. The lateral condensation method with the use of a sealer was employed for more effective sealing during obturation. Additionally, apical root resection was carried out, and the depth of dye diffusion was evaluated to assess the effectiveness of endodontic sealers in the process of obturation.

In the second part of the study, experimental procedures were conducted on eight rabbits, following contemporary standards and methodologies. After general anesthesia and preparation of hard tooth tissues, the pulp coronal portion was amputated, and iRoot BP Plus endodontic sealer was applied for obturation. Subsequently, a 6-week observation period was conducted to monitor the processes of tissue regeneration and restoration following pulp removal, enabling the evaluation of the impact of different endodontic sealers on regenerative processes.

The study included the following sample sizes for each group of endodontic sealers:

Trioxident group - *n* = 10 teeth.

Bioceramic-based sealer (Bioceramic Sealer) group - *n* = 10 teeth.

Rutdent group - *n* = 10 teeth.

Mineral trioxide aggregate (MTA) group (ProRoot MTA) - *n* = 10 teeth.

iRoot BP Plus group - *n* = 10 teeth.

Additionally, a control group consisting of 50 teeth was used, where no endodontic sealer was applied (placebo or other material).

Furthermore, in the animal model (rabbits), there were two groups:

Experimental group - *n* = 8 rabbits.

Control group - *n* = 8 rabbits.

The control group consisted of teeth that underwent endodontic therapy but were not treated with any endodontic sealer. This approach allowed for a comparison of the results obtained in the groups where different endodontic sealers were applied to those teeth where no endodontic sealer was used. By doing so, specific effects associated with each of the ddontic sealers could be identified and analyzed.

In the context of experiments involving rabbits: A control group was established using rabbits that underwent pulp amputation, but instead of an endodontic sealer, a placebo with no tissue-regenerative properties was applied. Comparing the experimental groups to the control group allowed for the identification of specific effects of iRoot BP Plus and its influence on tissue regeneration processes in comparison to other alternatives. Rabbits possess continuously growing and wearing permanent teeth, making them suitable models for studying dental processes. Researchers can investigate both pathological conditions and tissue healing and regeneration processes using rabbits. Furthermore, rabbits exhibit similarities to humans in dental anatomy and physiology, enabling researchers to conduct studies on rabbits and obtain results that may have greater applicability to human dentistry. Despite certain limitations, such as continuous tooth wear, studies involving rabbits can provide valuable data and insights into various dental procedures and biomaterials.

The experiments were conducted by experienced researchers possessing professional knowledge and skills in the fields of dentistry and surgery. These researchers successfully performed procedures involving endodontic therapy, canal obturation, and apical resection in the first part of the study. Additionally, they conducted tooth preparation and utilized iRoot BP Plus for pulp capping in rabbits during the second part of the experiment. Adhering strictly to ethical principles and international standards, the researchers ensured the reliability and trustworthiness of the study’s findings.

The primary objective of the animal experiment was to investigate the effects of various endodontic sealers, particularly iRoot BP Plus, on regeneration processes following pulp amputation. The study aimed to assess the ability of these materials to promote both the sealing and regeneration of dental tissues in experimental rabbits after pulp removal. Through this research, scientists sought to comprehend the potential of iRoot BP Plus in stimulating tissue regeneration and its effectiveness in restorative procedures post-pulp amputation. The results obtained from the animal experiments may offer valuable insights for further research and potential applications in dentistry, particularly in the realms of endodontic and regenerative procedures. It is essential to note that animal experiments are frequently conducted before human clinical trials to evaluate safety and efficacy before potential human applications.

The study conducted with animals adheres to international ethical standards. Histological studies on rabbits were conducted by the Helsinki Declaration of the World Medical Association. The study protocol was approved by the Ethics Committee of Beijing Medical University (Protocol No. 456). Data analysis was performed in Past v. 4. The statistical significance of differences was determined using the Kruskal-Wallis test. The pairs of independent samples were compared using the Mann-Whitney test. A normality test: Determining the normality of a distribution allows assessing the degree to which data conform to a normal distribution. This is essential to appropriately apply subsequent statistical tests that assume data normality. In cases where the data deviates from a normal distribution, alternative statistical methods can be employed.

Kruskal-Wallis test: This non-parametric test is employed to compare values among three or more independent groups of data when the data do not conform to a normal distribution or when other assumptions for applying parametric tests, such as one-way analysis of variance (ANOVA), are not met. In this study, the Kruskal-Wallis test may have been used to compare the results among the five different types of endogermatics.

Mann-Whitney U test: This non-parametric test is used to compare values between two independent groups of data. It is sensitive to differences in medians and does not require assumptions of data normality. The Mann-Whitney U test may have been used to compare independent groups of data within each type of endodermic in the present study.

Differences were considered significant at *p* ≤ 0.05.

## Results

### Marginal permeability

**Fig. 2:**
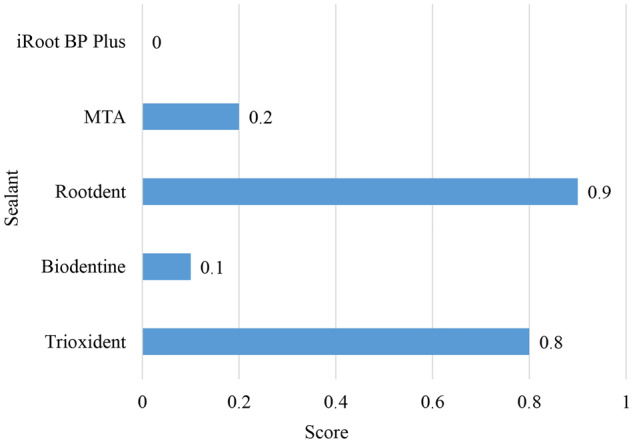
Comparison of dye penetration among five different biomaterials for pulpitis and perforation treatment

The endodontic biomaterials in question showed different degrees of dye penetration (Fig. 2). The pairwise comparison of independent samples also revealed significant differences. The results are depicted in Fig. 2 and Table 1. The control group demonstrated a statistically significant absence of dye penetration.

**Table 1:** Pairwise comparison of dye penetration depths (Mann-Whitney test)

	Trioxident	Biodentine	Rootdent	МТА	iRoot BP Plus	Kruskal-Wallis test
Trioxident		0.008509^a^	0.7198	0.02819^a^	0.001826^a^	0.00014
Biodentine	0.008509^a^		0.002593^a^	0.5828	0.3681	
Rootdent	0.7198	0.002593^a^		0.009294^a^	0.000532^a^	
МТА	0.02819^a^	0.5828	0.009294^a^		0.1675	
iRoot BP Plus	0.001826^a^	0.3681	0.000532^a^	0.1675		

^a^represents a statistically significant difference

**Fig. 3:**
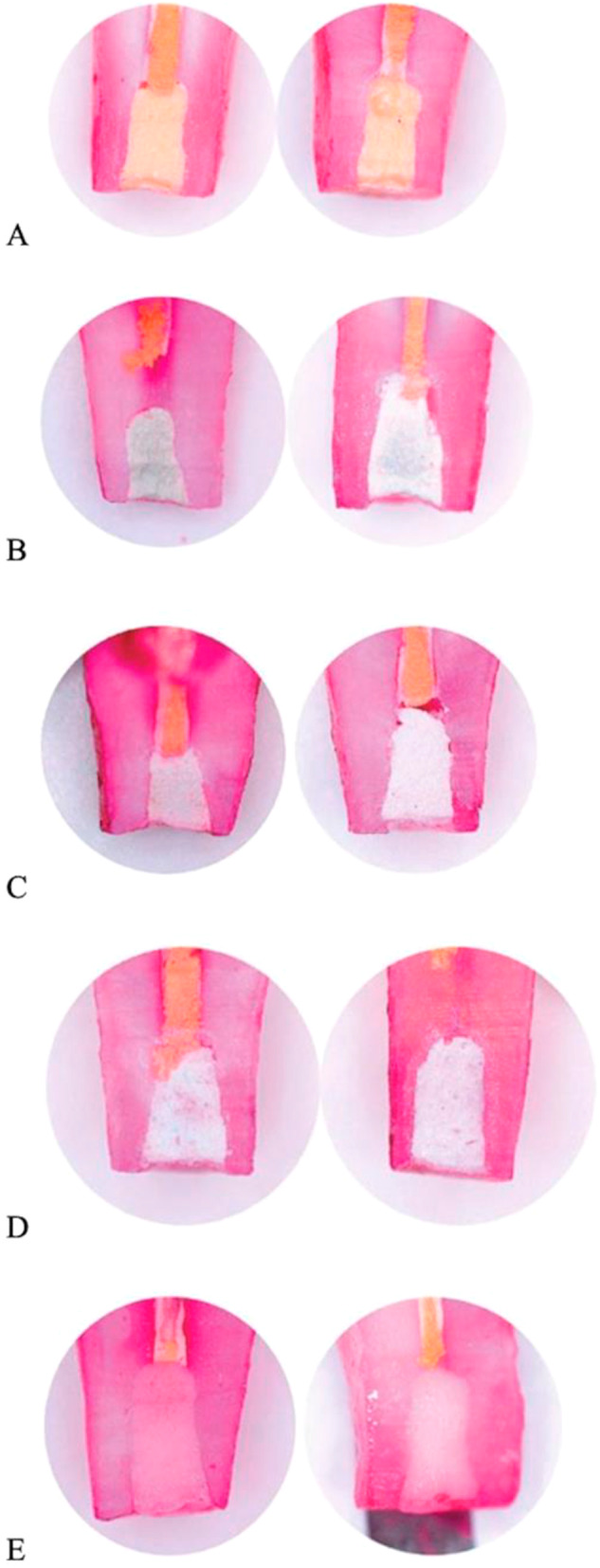
Dye penetration depths: (**A**) – iRoot BP Plus, (**B**) – MTA, (**C**) – Biodentine, (**D**) – Trioxident, (**E**) – Rootdent. Note: **A**, **B** – No dye penetration; **C**, **D** – Marginal dye penetration along the filling-tooth interface; **E** – Complete dye penetration along the filling-tooth interface and into the filling material

iRoot BP Plus showed the lowest dye penetration score in all cases with no dye penetration. This finding suggests that iRoot BP Plus has high-insulating properties. MTA and Biodentine were less effective, having 2–1 cases of dye penetration along the filling-tooth interface, respectively. The remaining two biomaterials were the least effective. Trioxident had 6 cases of dye penetration along the interface and 1 case of dye penetration into the filling material. Rootdent had 7 cases of marginal penetration and 1 case of complete penetration. Since Trioxident and Rootdent failed to keep the dye from penetrating deep inside them, these two biomaterials can be deemed ineffective. The results of the dye penetration test are depicted in Fig. 3.

### Potential use of iroot BP plus in dental pulp removal

The surgical interference resulted in the emergence of a keratinized stratified squamous epithelium.

**Fig. 4:**
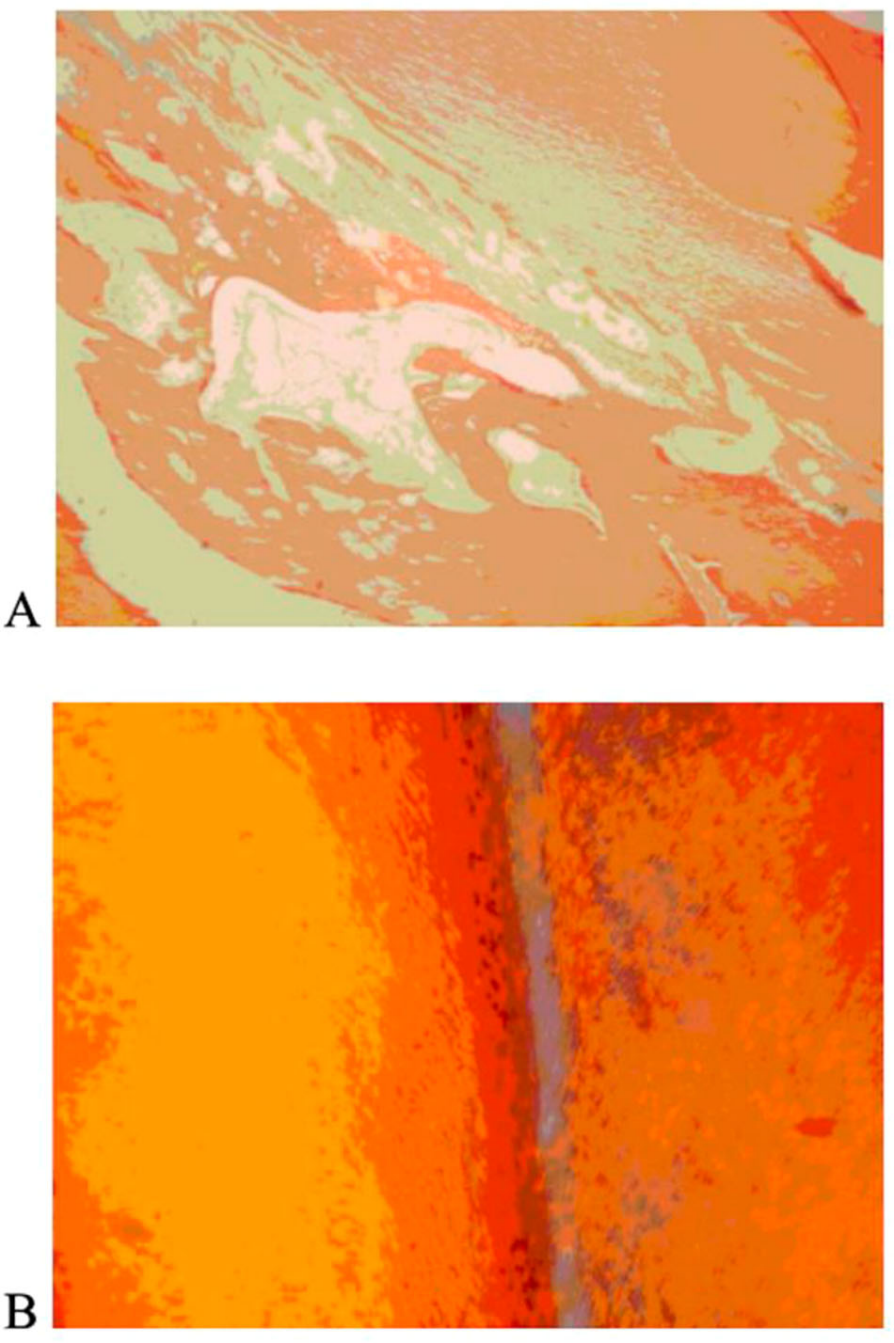
Microscopic evaluation of the treated area after dental pulp amputation (**A**) and dentinal tubule preservation in enamel (**B**) at 200x magnification, stained with Van Gieson and Mallory’s Dyes, respectively

Histological findings showed normal stratified epithelium layers with slight basal layer proliferation. The dentin layer in the treated area had no protrusions and presented a linear appearance (Fig. 4A). Dentinal tubules retained their radial structure with small interglobular spaces. Enamel-dentin boundary exhibited a linear interface, and enamel tufts were observed in the border area (Fig. 4B). Distal dentinal tubules in the enamel zone remained intact but slightly loosened near the dentin. Striated dentin had channels radiating through a ground substance, covered by unstructured enamel at the crown and cementum on the root. Intertubular dentin near the injury showed homogeneity, fragmentation, and no dentinal tubules, while peritubular dentin showed curvature within a homogeneous layer. The intertubular dentin contained globular spaces due to uneven mineralization. Predentin near the odontoblasts in the dentin-pulpal border zone was ribbon-like with dentinal tubules. The dentin adjacent to the pulp showed no deformation, except for one case. Uneven deposition of secondary dentin was observed. High tissue vascularization was present with numerous vessels and lymphatic ducts. Predentin in the filling-tissue border zone was thin, and dentinal tubules were slightly deformed. Connective tissue fibers and newly formed vessels were observed. Odontoblast cells exhibited vacuolar hydropic degeneration.

For the control group, the following results were obtained: Absence of tissue regeneration: In the control group, where a material lacking tissue regeneration-stimulating properties was applied, it was observed that the processes of tissue regeneration and healing after pulp amputation were reduced or absent. This may lead to a limited organism’s capacity to recover damaged tissues and form new cells.

Absence of neovascularization: Tissue regeneration stimulation is typically accompanied by the formation of new capillaries to supply oxygen and nutrients to the tissues. In the case of using a material lacking regenerative properties, there was no active formation of new capillaries at the site of pulp amputation.

Delayed wound healing: The absence of tissue regeneration stimulation resulted in delayed wound healing after pulp amputation. This may indicate that the wound remains open and susceptible to potential inflammation and infection.

Inflammatory reactions: In the absence of tissue regeneration stimulation, more pronounced inflammatory reactions were observed.

Based on the results of the marginal permeability test, iRoot BP Plus demonstrated high efficiency in preventing dye penetration, suggesting moderate activity of inflammatory cells. On the other hand, Trioxident and Rootdent showed insufficient effectiveness, allowing dye penetration into the material, and indicating increased activity of inflammatory processes.

The histological data obtained from the surgical intervention using iRoot BP Plus indicate the occurrence of aseptic inflammation, accompanied by the formation of connective tissue bridges, supporting regenerative processes. In contrast, the control group, where a material without tissue-regenerative stimulating properties was used, revealed a lack of tissue regeneration, absence of neovascularization, delayed wound healing, and more pronounced inflammatory reactions, suggesting higher activity of inflammatory cells in the absence of regenerative stimulating properties.

Thus, the data support the idea that iRoot BP Plus may contribute to the activation of aseptic inflammation while simultaneously supporting tissue regeneration. The limited dye penetration into the iRoot BP Plus sealer, compared to other sealers, may be associated with its high sealing ability, determined by the unique features of its chemical structure. IRoot BP Plus may have a formula or molecular network that creates a denser and impermeable structure, preventing the dye from penetrating its depth. Additionally, it is worth considering that iRoot BP Plus showed the lowest dye penetration scores in all cases, emphasizing its effectiveness in preventing the penetration of moisture and other substances into the treated teeth.

These data suggest that when used as biomaterials during pulp removal, iRoot BP Plus facilitates the onset of aseptic inflammation. In parallel, it supports the formation of connective tissue bridges, promoting the regeneration processes.

## Discussion

Today, biomaterials are an indispensable element in a dentist’s work, for bioactive ceramics exhibit good compatibility with the surrounding tissues and excellent adhesion properties [25].

The study [26] focuses on evaluating the potential risks of undesirable biological effects associated with the interaction of these materials before their integration into clinical practice. The authors emphasize that the results of biocompatibility assessments depend not only on the materials themselves but also on testing methods due to the diversity of effects and numerous variables. The structured assessment of materials includes four phases: general toxicity, irritation of local tissues, preclinical trials, and clinical evaluations. The paper also discusses various screening methods for assessing biocompatibility and underscores the need to understand their advantages and limitations for the accurate interpretation of results. The authors highlight recent scientific advancements introducing new materials into endodontics, such as nanomaterials, genetic therapy, and biomaterials for tissue engineering. They emphasize the importance of preliminary clinical testing, adherence to international standards (e.g., ISO 10993, ISO 7405, and ISO 14155-1), and careful compliance with safety assessment methods for the successful integration of new materials into medical practice [26].

The present study highlights the same advantages, although some endodontic biomaterials appeared to be more effective in terms of adhesion than others. Bioceramics also benefit from high pH values (antibacterial properties) and short setting time in the presence of water. Further research is currently needed to identify the properties of multiple bioceramic endodontic biomaterials that can be found on the market, for their quality does not always correspond to the level declared by the manufacturer [2, 27]. This problem is evident from the current comparative analysis of 5 different endodontic biomaterials: some of them were proven to have better air tightness, while others (Rootdent) had leakages. Future research should compare the main endodontic biomaterials with each other by reviewing the state-of-the-art literature.

MTA is used for root-end filling, repair of root perforations, and pulp capping [28]. Until 2002, there was only one MTA-based biomaterials on the market. Subsequently, the material was modified to ProRoot MTA. According to many researchers, the distinctive property of MTA is its long setting time (165 min, on average). The porosity of the hardened mass thus depends on the water-to-powder ratio [29]. Its compressive strength increases over time from 40 MPa after a day to 66 MPa after 3 weeks. In addition, its flexural strength also tends to increase within a span of 1 day [30]. At the same time, MTA has relatively low adhesion strength compared to other biomaterials. Due to its high pH (up to 12.5), it has a pronounced antibacterial effect on at least six bacterial species [31]. Moreover, MTA promotes the expression of cytokines and favors cell migration. The drawbacks include the need for complex manipulations, high cost, the absence of a ready-to-use mixture, and the difficulty of removing root canals from the lumen [32].

Biodentine belongs to the second generation of bioceramic endodontic biomaterials. It is a calcium-silicate-based material biomaterial with properties that are somewhat superior compared to those of MTA. For instance, its final sitting time is significantly lower (45 min), while the initial setting time is 9–12 min [30, 33]. The compressive strength of biodentine reaches 100 MPa in the first hour and triples within 1 month, significantly outperforming the MTA. The elastic modulus of biodentine is close to that of dentin (about 19 MPa), whilst the flexural strength is within 35 MPa after 2 h [34]. Among the advantages of biodentine is the ease of use and better physical/chemical properties compared to MTA. Biodentine can be used for composite restorations and indirect pulp capping. It is less prone to bacterial contamination than MTA [28]. Biodentine is commonly used in standard dental procedures, such as root perforations, apexification, and retrograde fillings. Yet, it is not recommended for use in tooth reconstruction in cases where a significant portion of the tooth crown is missing. Also, it is not applied in the repair of anterior teeth and calcified structures damaged by irreversible pulpitis [2].

iRoot BP Plus is intended for root canal perforations. Its initial setting time is 10 min, but it can take up to 4 h for the material to fully harden [35–37]. Its advantages include ease of use, existence in a ready-to-use form, antibacterial activity, low tissue toxicity, and the ability to mineralize dentinal structures in the tooth [38]. Upon contact with liquids, iRoot BP Plus generates a layer rich in silicon dioxide where hydroxyapatite is formed [36].

In a study [39], it was demonstrated that MTA and Biodentine exhibited similar success rates (100% and 95%, respectively). The research was conducted on a sample of 20 patients and indicated that Biodentine showed a high therapeutic effect, with mild inflammation, making it a potentially safe biomaterial for direct pulp capping. In a study [40], the results of the effects of MTA and tricalcium silicate cement (Biodentine) on inflammatory reactions and dentin regeneration in Wistar rat models were presented. This investigation revealed that Biodentine could lead to pulp calcification, although both biomaterials demonstrated low levels of inflammation in adjacent tissues. The outcomes of replantation of upper right incisors in 16 rats using MTAF and CH biomaterials showed no significant differences in inflammatory reactions and resorption, and all replanted teeth survived [41]. Therefore, both our study and the studies of colleagues have demonstrated that different biomaterials may exhibit varying effectiveness, being superior or inferior in different parameters when compared to their competitors. The present study shows that it not only has excellent sealing ability, but these biomaterials can also accelerate regeneration due to low cytotoxicity. Therefore, iRoot BP Plus can be recommended as a high-quality endodontic biomaterial for pulp removal and root canal fillings.

## Conclusions

iRoot BP Plus showed the best dental outcomes with no marginal dye penetration (score 0). Several cases of marginal penetration were seen with Biodentine and MTA; yet, the number of such cases was insignificant, suggesting that these two biomaterials are slightly worse but as effective as iRoot BP Plus. Trioxident and Rootdent exhibited the worst sealing performance (0.8–0.9 points), with 9–8 cases of marginal dye penetration and 2–1 cases of complete dye penetration. Based on these results, iRoot BP Plus can be recommended as a high-quality biomaterial for retrograde apical root filling, along with Biodentine and MTA.

The aseptic inflammation accompanying the regeneration processes seems to have ended 6 weeks after the pulp was removed. At the same time, some dentin fragments were found to be encapsulated, whilst the necrotic foci became isolated from the healthy pulp tissue. The intense formation of new vessels and lymphatic ducts was detected. Based on the presented data, iRoot BP Plus can be recommended as a potentially high-quality biomaterial for retrograde apical filling, on par with Biodentine and MTA. However, for more confident recommendations and further assessment of long-term effects, additional clinical research involving a larger number of patients is necessary. Subsequent investigations could focus on evaluating the long-term effects of this endodontic biomaterial and its application in various conditions. Additionally, it is essential to establish the appropriate indications and circumstances for the use of iRoot BP Plus.

Future research can focus on evaluating the long-term effects of these endodontic biomaterials and expand to other sealing agents. In addition, it is necessary to justify when iRoot BP Plus can be used. Finally, this study is restricted to animal experiments, indicating the need for analogous investigations involving human participants to complement the findings.

## Data Availability

Data will be available on request.
